# Sensing, feeling and consciousness

**DOI:** 10.1098/rstb.2023.0243

**Published:** 2024-08-26

**Authors:** Antonio Damasio, Hanna Damasio

**Affiliations:** Brain and Creativity Institute and Department of Psychology, Dornsife College of Letters, Arts, and Sciences, University of Southern California, Los Angeles, CA 90089, USA

**Keywords:** consciousness, feeling, interoception, experience, subjectivity

## Abstract

Living organisms achieve homeostasis by using distinct mechanisms tailored to their physiological complexity. Unicellular organisms as well as plants, which are devoid of nervous systems, rely on covert sensing/detecting and equally covert responding mechanisms. Organisms with nervous systems rely on *overt* consciousness which is based on homeostatic feelings and the experiences and consequent subjectivity they generate.

This article is part of the theme issue ‘Sensing and feeling: an integrative approach to sensory processing and emotional experience’.

1. 

To a first approximation, sensing, feeling, and consciousness seem difficult to distinguish given that all three processes are concerned with maintaining life by securing homeostasis in a specific organism. But beyond the common goal of ensuring self-sustaining chemical operations within a body and thus making the continuation of life possible, each of the three processes is quite distinct and operates in organisms whose complexity is different as well.

Sensing is ubiquitous in nature. All single cell organisms survive thanks to sensing because when stimuli of varied sorts—chemical, mechanical—impinge on cellular membranes, they are detected by a variety of micro-physiological components and the cell generates a response. Bacteria go through their lives sensing and responding, as do plants, but neither plants nor bacteria have nervous systems to help them with the sensing and responding. Their sensing and responding is intelligent, from the standpoint of their lives, but neither plants nor bacteria *know* that they have sensed or responded, let alone that their lives are being made possible thanks to sensing/detecting and responding. They do not have explicit knowledge of what goes on inside their organisms, which means that they do not *represent* the premises of the problem they are solving and thus cannot be conscious of the operations that have just made their lives possible. To a naive observer they appear not only smart but well informed, and, well, conscious. But they are not. Their behaviors are indeed intelligent but one might say that their intelligence belongs to their species more than to the surviving individual; it is an intelligence ‘meant’ to save the exemplars of a species rather than a particular creature.

Neither plants nor bacteria have nervous systems and without nervous systems they have a limited possibility of *representing* the situations they are in and appreciating their predicaments. They lack the possibility of making maps and images of parts of their organism and literally *picturing* their situation. In the absence of representing such details they lack an essential means of *experiencing* any situation.

The strange knowledge we have just described in bacteria and plants is appropriately known as *covert* because it is perfectly hidden to the living creature that benefits from it. Another designation for it is *implicit* knowledge but perhaps the most adequate name is *non-conscious knowledge*. For creatures that lack a nervous system all knowledge is covert and non-conscious. Nervous systems are a passport to overt knowledge, beginning with *knowledge about the knowing creature itself*. It turns out that a significant part of the overtly represented knowledge is provided in the form of homeostatic feelings. Such feelings are the natural grounding of consciousness and open the way for *life regulated by choice and deliberation*, the very opposite of covert life regulation. For a comprehensive treatment of the distinction between covert and overt (conscious) life regulation, see *The Strange Order of Things*, [1].

The distinction we have just drawn between conscious and non-conscious living organisms is not universally accepted. For example, Reber [2] has argued that all cells, including prokaryotic archaea and bacteria, can be conscious, an idea that we have not accepted (see A. Damasio, 2019 in [2]; see also [3]). It is apparent that these single cells are equipped with intelligent devices that allow the complex navigation of their lives, so complex in fact that it even allows for social behaviours. Nonetheless, there is nothing in the Reber–Baluška conception of cellular life that might produce the explicit representation/images that we regard as essential for the processes of feeling and consciousness.

2. 

Understanding consciousness and explaining how it can come about has been one of the most puzzling enigmas in biology. From our perspective the difficulty has had two main causes. The first is the lack of precision with which the term consciousness is usually defined, which includes it not being defined at all and merely being presented as a problem whose definition must be obvious. The second cause comes from the manner in which the problem of consciousness tends to be situated, high in the scale of biological complexity, at the level of cognition or even higher. Our attempt to deal with the consciousness problem, on the contrary, begins far lower in that scale and places consciousness as a critical component of life regulation. In fact, in our definition of the phenomenon, *consciousness is a principal means for a living organism to experience the state of operations inside its body*. This is another way of saying that consciousness allows an organism to know about itself and permits that same organism to have a perspective—a *subjective perspective*—on itself and on the universe that surrounds it. That is precisely the sort of informed, knowledgeable ‘attitude’ that opens the way for organisms to regulate their lives deliberately and to leave behind and move away from the covert sensing and responding of creatures without nervous systems.

Should the above considerations appear unconventional, we need to add something even more surprising, namely, the strangely unrecognized status of homeostatic feelings as the *inaugural* events of consciousness itself. We are saying that each and all homeostatic feelings, from the most obvious—such as hunger, thirst, pain, well-being, malaise, desire—to the most subtle—such as the feelings of body temperature, breathing and cardiac function—is telling us, in no uncertain terms, that we *exist*, that we are faring well or not so well, and that we may need to act in order to obtain something that we lack and fare even better, e.g. food, drink, sex, temperate climate, or the right kind of love. Quite clearly, each and all of the homeostatic feelings we have just enumerated are important to the maintenance of life. No less importantly, however, each of them, either acutely or continuously, informs a mind that is naturally oriented to representing the surround of its organism, that there is a process of life going on within that same organism and that it can operate *better or worse* in the adventure of existence. In brief, spontaneously conscious feelings suggest to each organism what it should or might do next, such as to procure food or drink so as to maintain its life, for example, or look for safety or mates.

As we see it, the saga of consciousness begins with homeostatic feelings but takes the organisms so endowed quite far. The continuous fabric of feeling allows organisms to experience their own life process continuously; organisms are literally allowed to discover their own existence. And when organisms are also capable of generating images of their environment—for example, visual and auditory images—the combination of feelings with the images from their surround, produces a perspective of the organism relative to that surround. This literally constitutes a *subjective perspective*, where the ‘subject’ is the felt/experienced organism. In all likelihood, this is the biological origin of the *self* process.

In complex organisms, this novel and subjective perspective serves as an anchor to their extended cognitive capacities, which include: (a) recording of images in memory and ability to recall such images and manipulate them in a reasoning process; and (b) the capacity to translate any image in a language code and thus operate both at primary image level and verbal level.

The consciousness of humans and of other complex organisms is the result of combining the process of homeostatic feeling with that of exteroceptive images. Such a combination requires a nervous system, to be sure, but it also requires the internal, non-neural machinery of a complex organism. The feeling process, as we see it, is the consequence of a partnership between a sector of the nervous system—the interoceptive sector—and the non-verbal interior of the organism responsible for the life process (a hypothesis discussed in [4] and [5]).

In conclusion, consciousness arises in organisms endowed with nervous systems but *not* from the nervous system alone. Consciousness is the result of a co-mingling between a body's visceral interior *and* its nervous system. The partnership produces feelings, which reflect, at each moment, the manner in which life is being pursued: efficiently, and perhaps with a flourish, or inefficiently and with the risk of disease.

3. 

Explaining consciousness in terms of the emergence of homeostatic feelings promises a solution to the issue of what consciousness is and, no less importantly, it opens the possibility of explaining how consciousness is generated.

As we see it, homeostatic feelings depend on two principal features of the nervous system. The first is the specific nature of the neurons in the *interoceptive* nervous system, which is the system charged with signalling from the body's interior—its viscera, its respiratory and circulatory systems, its endocrine and immune systems, its ‘flesh' in the ample sense of the word—to the central neural components which orchestrate the regulation of life. The interoceptive neurons belong to an old evolutionary vintage and their operation is distinct in every respect: their axons are poorly myelinated or not myelinated at all, which means that they are poorly insulated, can be affected by a variety of molecules present in their surround and their activity is not independent of the non-neural flesh that surrounds them. They also operate very slowly, and as if these distinctions were not enough, interoceptive neurons in peripheral nerve ganglia as well as in nuclei of the central nervous system are at the mercy of molecules in the blood circulation. This is because those regions are *not* protected by the blood–brain barrier, an insulating feature that in evolutionarily modern sectors of the nervous system separates the operations of the nervous system from those of the non-neural compartments of the organism and thus insures the relative independence of the former. There is no such independence for the interoceptive nervous system, peripheral as well as central, and the result is that neural and non-neural operations *co-mingle*. In our judgement this is *the* main feature responsible for homeostatic feelings, the physiological feature that most closely accounts for experiences and thus for consciousness (see [6] and [7]).

One other feature of interoception is critical for the generation of homeostatic feelings but is systematically overlooked: in any complex organism, *the nervous system is entirely located within the perimeter of the body*. This easily overlooked arrangement permits a unique functional dialogue between ‘the body’ and ‘the nervous system’. For example, when the body signals the presence of a serious injury to the nervous system, the nervous system can not only produce the appropriate feeling—pain—but also respond to the presence of damage by enacting chemical remediation and reducing the feeling of pain itself. This arrangement is unique. Notably, there is nothing comparable in the relation between the nervous system and the universe that surrounds our organisms.

4. 

In conclusion, the emergence of consciousness in evolution can be reasonably tied to the evolutionary development of homeostatic feelings whose presence is responsible for generating continuous experiences of the living organism and supports the process of subjectivity. Homeostatic feelings are essential to the process of consciousness encountered in vertebrates, in which cognitive processes alone cannot explain consciousness.

## Data Availability

This article has no additional data.
